# Impact of desertification on soil and plant nutrient stoichiometry in a desert grassland

**DOI:** 10.1038/s41598-019-45927-0

**Published:** 2019-07-01

**Authors:** Hui An, Zhuangsheng Tang, Saskia Keesstra, Zhouping Shangguan

**Affiliations:** 1 Breeding Base for State Key Laboratory of Land Degradation and Ecological Restoration in Northwest China, Ningxia University, Yinchuan, Ningxia 750021 China; 2 State Key Laboratory of Soil Erosion and Dryland Farming on the Loess Plateau, Northwest A&F University, Yangling, Shaanxi 712100 China; 3 Soil Physics and Land Management Group, Wageningen University, 6708 PB Wageningen, The Netherlands; 4 Civil, Surveying and Environmental Engineering, The University of Newcastle, Callaghan, 2308 Australia

**Keywords:** Grassland ecology, Grassland ecology

## Abstract

Grassland degradation resulting from desertification often alters the carbon (C), nitrogen (N) and phosphorus (P) cycles within grassland ecosystems. To estimate the effects of desertification on the C, N, and P concentrations and C:N:P stoichiometry of plants and soil, we examined C, N, and P concentrations in plant tissues (leaves, roots and litter) and soil across five degrees of desertification in the desert grassland of Ningxia, China (control, light, moderate, severe and very severe desertification stages). The C, N, and P concentrations and C:N:P stoichiometry of the leaves, roots and litter differed among the different desertification stages. Desertification resulted in opposing trends between the leaf N concentration and leaf C:N ratio. With the exception of the very severe desertification stage, the leaf N:P ratio decreased over the process of grassland desertification. The soil C, N, and P concentrations and soil N:P and C:P ratios decreased significantly along the grassland desertification gradient. In contrast, the soil C:N ratio remained relatively stable during desertification (10.85 to 11.48). The results indicate that desertification is unfavourable to C and N fixation and has a negative effect on the ecosystem structure and function of desert grassland.

## Introduction

Grassland desertification, the primary form of land degradation in northern China, is defined as the degradation of grasslands in arid and semiarid regions resulting from various factors, including climate change and human activity. Desertification has caused major environmental and socioeconomic problems in many arid and semiarid areas of the world^1^. It causes soil degradation and severely reduces potential land productivity^2–4^, which causes degradation of the ecosystem and its associated ecosystem services. Therefore, the economic development of the region is also under threat. In 2015, the UN adopted the Sustainable Development Goals (SDGs), and many of them are connected to soil functions^5^. This shows the importance of understanding soil quality and the processes affecting soil quality for sustainable economic development and topics such as climate change mitigation, water resource management and biodiversity. In addition, global change studies have increasingly focused their attention on desertification in recent years because of its effects on regional and global climate change^2,6–9^. Desertification has been described primarily in terms of its effects on vegetation and soils. Grassland desertification is marked by the replacement of native by exotic species. Thus, desertification results in changes in vegetation composition, pattern and structure^3,10^. Soil organic carbon (SOC), nitrogen (N) and phosphorus (P) are often observed to decrease with land desertification^3,11^. However, the impact of desertification on the plant and soil C:N:P stoichiometry in desert grasslands remains unknown.

Ecological stoichiometry, which plays vital roles in the study of vegetation composition, ecosystem functioning, and nutrient limitation^12–14^, has greatly improved our understanding of terrestrial ecological dynamics and processes. In recent years, several studies have used regional or global-scale patterns in plant stoichiometry to predict vegetation composition and dynamics and nutrient limitation^15–18^. Researchers have also focused on how the balance among soil C, N and P concentrations may regulate vegetation patterns^19^. The soil C:N:P ratio directly reflects soil fertility and indirectly indicates plant nutritional status^20,21^. Changes in soil organic C and total soil N and P concentrations inevitably result in variation in nutrient stoichiometric relations^22,23^. Variation in climatic, soil and plant physiological characteristics (i.e., plant growth, metabolism and life history traits) are considered to be the primary factors influencing plant C:N:P stoichiometry^24^. The variation in plant C:N:P stoichiometry in relation to soil N availability was analysed by Méndez and Karlsson^25^, and they found a significant impact of soil N on plant N:P stoichiometry. However, some other studies found that only some nutrient ratios were influenced by changes in soil nutrients^26^, or no relationship was found between soil and plant C:N:P stoichiometry^27^.

In the biogeochemical cycles of grassland soils affected by desertification, inevitable changes in the C, N and P cycles were found as a result of the effects of desertification on soil properties. Most studies have focused on the influence of desertification on soil organic C and N^2,28,29^. However, there is little knowledge regarding the C:N:P stoichiometry of plants and soil in relation to grassland desertification. Changes in the soil C:N ratio showed opposite trends in the sandy grasslands of Inner Mongolia and the alpine meadow of the Qinghai-Tibetan Plateau during desertification^2,29^. The soil C:N ratio decreased in the sandy grassland of Inner Mongolia during desertification but increased in the alpine meadow of the Qinghai-Tibetan Plateau. The soil C:N ratio increased during the process of desertification due to the resultant reductions in soil organic C and N^29^.

Ningxia is located in the transitional zone between the arid and semiarid regions of northwest China and is surrounded by the Mu Us Desert, the Tengger Desert, and the Ulan Buh Desert. The desertification process has been significantly reversed due to the implementation of some ecological engineering measures (i.e., the Grain for Green Project and region-wide grazing exclusion)^30^. However, the harsh natural environmental conditions and low socioeconomic status of the population have led to the development of ecologically fragile sandy areas. Therefore, the trend of desertification, officially described as “overall reversal but partial deterioration”, still exists^31^. Changes of vegetation composition, grassland productivity and soil physical and chemical properties during grassland desertification processes have been described in arid and semiarid regions^3,32–34^. However, studies on the C:N:P stoichiometry of plants and soil during grassland desertification processes are very lacking. Previous studies on soil stoichiometry have mainly focused on the C:N ratio in response to the process of grassland desertification^2,29^. Therefore, few studies have focused on the relationships between nutrient contents and the stoichiometric ratios of soil and plants. The objective of this study was to determine how plant and soil C, N, and P and their stoichiometric ratios vary across grassland desertification stages. Our study addressed the following questions: (1) How do plant nutrient concentrations and stoichiometry change in different plant tissues (leaf, litter, root) in different stages of the desertification process? (2) What are the patterns of the soil C, N, and P concentrations and C:N:P stoichiometry during the desertification process? (3) What are the relationships of C, N, and P concentrations and C:N:P stoichiometry between plant tissues and soil in desert grassland ecosystems?

## Results

### Soil C, N, and P and stoichiometry response to desertification

Grassland desertification resulted in a significant reduction in soil C, N, and P concentrations and the soil N:P and C:P ratios (Table 1, *P* < 0.05). The soil C, N, and P concentrations and N:P and C:P ratios were greater in the potential desertification stage (PD) than in the other desertification stages. Along the grassland desertification gradient, the soil C, N and P concentrations ranged from 0.23 to 0.08%, 0.023 to 0.006% and 0.041 to 0.036%, respectively. The soil N:P and C:P ratios ranged from 0.48 to 0.18 and 6.12 to 1.89 and decreased by 63% and 70% from the PD stage to the VSD stage, respectively. In contrast, the soil C:N ratio ranged from 10.85 to 11.48 and did not differ significantly among the different desertification stages.

**Table 1:** Effects of desertification on soil C, N, P concentrations and stoichiometric ratios.

Desertification stage	C (%)	N (%)	P (%)	C:N ratio	N:P ratio	C:P ratio
PD	0.23 ± 0.01a	0.023 ± 0.002a	0.041 ± 0.001a	11.08 ± 0.07a	0.48 ± 0.07a	6.12 ± 0.20a
LD	0.18 ± 0.02b	0.015 ± 0.001b	0.040 ± 0.001a	11.22 ± 1.89a	0.31 ± 0.08b	4.34 ± 0.31b
MD	0.12 ± 0.03c	0.009 ± 0.001c	0.037 ± 0.001b	10.85 ± 0.04a	0.23 ± 0.04c	2.90 ± 0.39c
SD	0.10 ± 0.02cd	0.006 ± 0.001d	0.037 ± 0.001b	11.48 ± 0.78a	0.16 ± 0.02d	2.51 ± 0.27c
VSD	0.08 ± 0.02d	0.006 ± 0.001d	0.036 ± 0.002b	11.04 ± 0.91a	0.18 ± 0.02d	1.89 ± 0.14d
*P*	<0.05	<0.01	<0.01	NS	<0.01	<0.01

Soil nutrient concentration and stoichiometric ratios were measured in five different desertification stages: potential desertification stage (PD), light desertification stage (LD), moderate desertification stage (MD), severe desertification stage (SD), very severe desertification stage (VSD). Values represent treatment means ± standard deviation.

### Plant C, N, and P concentration response to desertification

**Figure 1:**
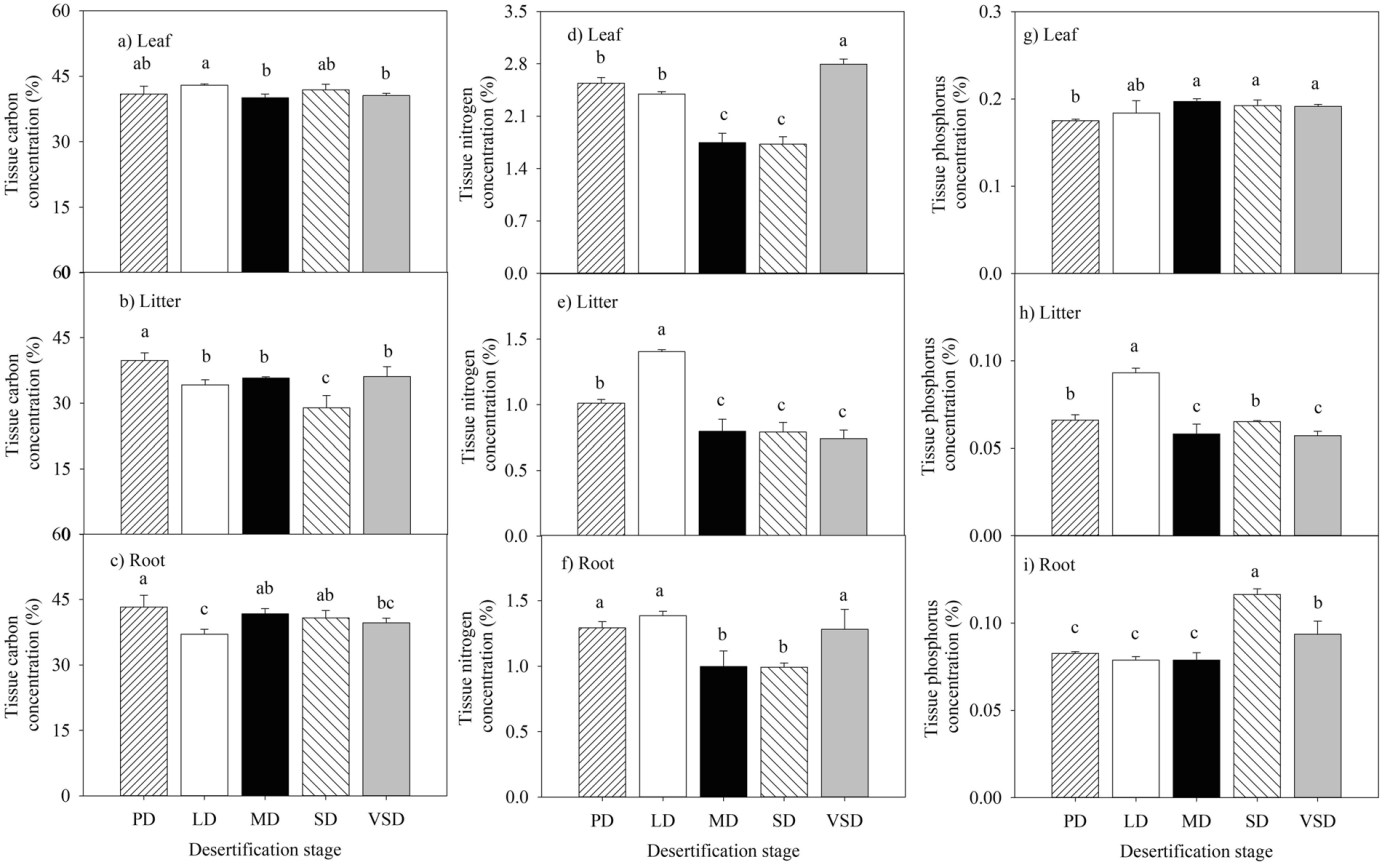
Response of C, N and P concentrations of plant tissue (leaf, litter and root) to desertification. Plant C, N and P concentrations were measured in five different desertification stages: potential desertification stage (PD), light desertification stage (LD), moderate desertification stage (MD), severe desertification stage (SD), very severe desertification stage (VSD). Different lowercase letters indicate differences at *P* < 0.05. Values represent treatment means ± standard deviation, n = 3.

With the exception of leaf C concentration, grassland desertification had a significant influence on the plant litter and root C concentrations (Fig. 1a–c; Table 2, *P* < 0.05). Plant litter C concentrations were greater in the PD stage than in the other desertification stages, but plant root C concentrations were greater in the PD stage than in the LD stage. The plant tissue (leaf, litter, and root) N and P concentrations were significantly influenced by grassland desertification (Fig. 1d–i; Table 2, *P* < 0.05). The plant leaf N and P concentrations were lower in the PD stage than in the VSD stage. The plant litter N and P concentrations were greater in the LD stage than in the other desertification stages. Plant root N and P concentrations were lower in the MD stage than in the VSD stage.

**Table 2:** ANOVA results comparing carbon (%C), nitrogen (%N), phosphorus (%P), the ratios carbon to nitrogen (C:N ratio), nitrogen to phosphorus (N:P), and carbon to phosphorus (C:P) of plant tissue (leaf, litter, root) in different desertification stages.

Plant tissue	% C	% N	% P	C:N ratio	C:P ratio	N:P ratio
Leaf	F = 3.26	F = 94.02^**^	F = 4.38^*^	F = 54.05^**^	F = 5.57^*^	F = 96.96^**^
Litter	F = 13.2^**^	F = 59.82^**^	F = 58.9^**^	F = 47.41^**^	F = 1.24^**^	F = 13.17^**^
Root	F = 5.90^*^	F = 11.94^**^	F = 42.29^**^	F = 17.61^**^	F = 95.81^**^	F = 28.31^**^

The level of significance with *P < 0.05, **P < 0.01.

### Plant C:N:P stoichiometric ratio responses to desertification

**Figure 2:**
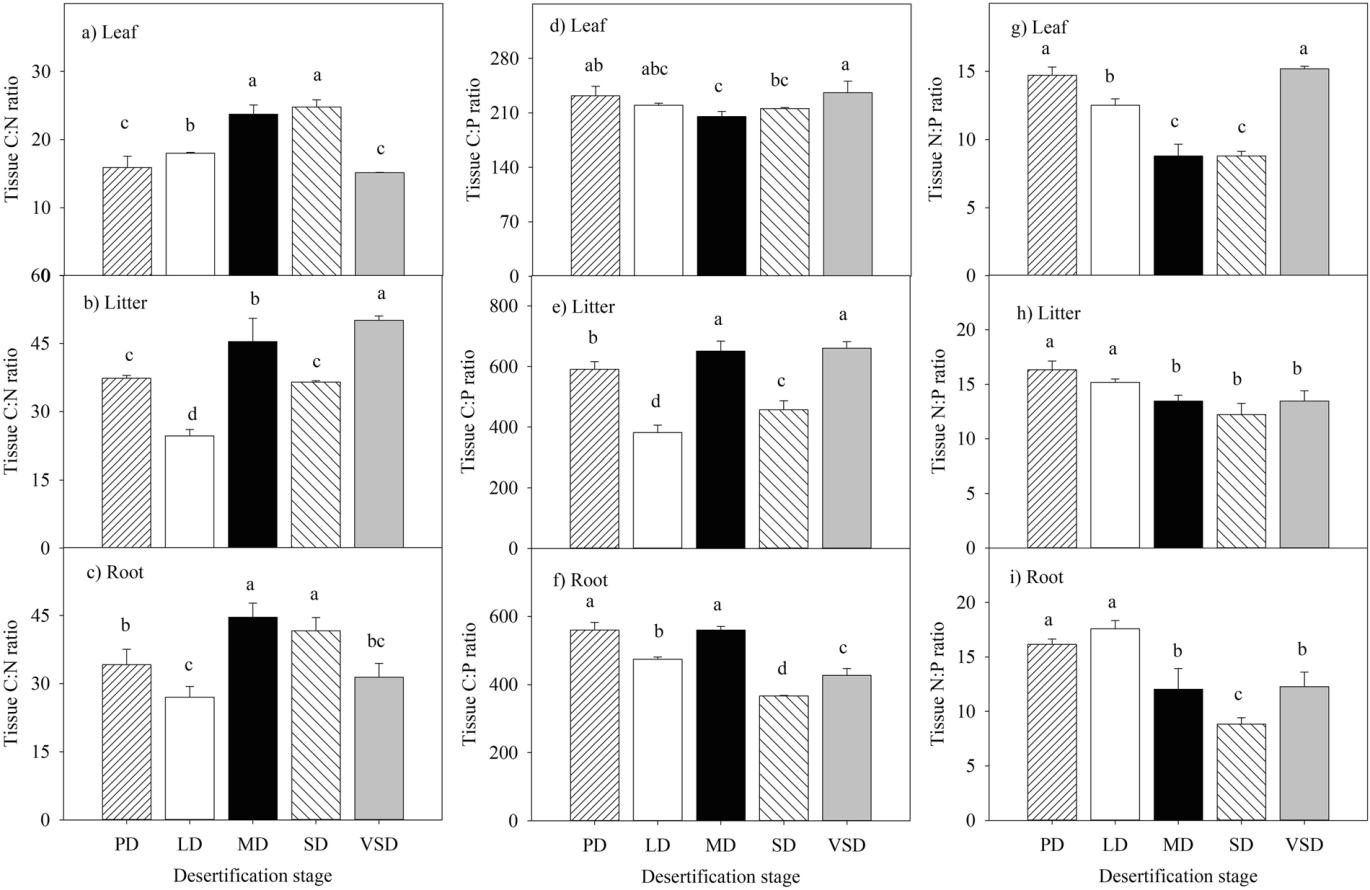
Response of C:N:P stoichiometric ratio of plant tissue (leaf, litter and root) to desertification. Tissue nutrient ratio were measured in five different desertification stages: potential desertification stage (PD), light desertification stage (LD), moderate desertification stage (MD), severe desertification stage (SD), very severe desertification stage (VSD). Different lowercase letters indicate differences at *P* < 0.05. Values represent treatment means ± standard deviation, n = 3.

Grassland desertification had a significant effect on the C:N, N:P, and C:P ratios of the leaf, litter and root (Fig. 2; Table 2, *P* < 0.05). The C:N and C:P ratios of the plant litter and root were lower in the LD stage than in the MD stage, while the N:P ratio was greater in the LD stage than in the MD stage. During the desertification process, the plant litter and root N:P ratios decreased by 25.3% and 45.3%, respectively. The leaf C:N ratio was lower in the VSD stage than in the MD and SD stages, while the leaf C:P and N:P ratios were greater in the VSD stage than in the MD and SD stages.

### The plant-soil relationships of C, N, and P concentrations and C:N:P stoichiometry

**Figure 3:**
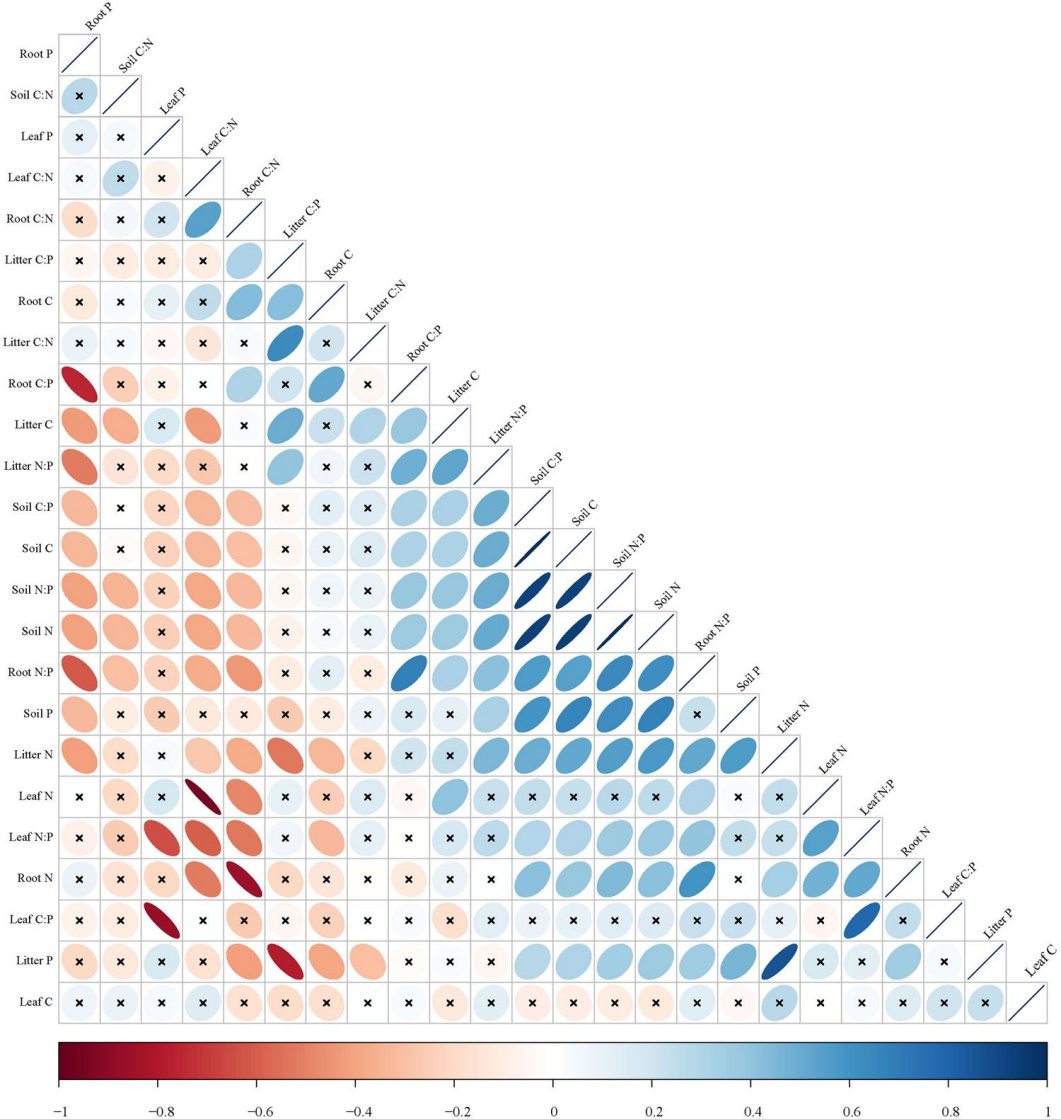
Correlation matrix among nutrient and stoichiometry of plant leaf, root, litter and soil. N = 10. Note: ‘×’ indicates correlation is non-significant (*P* > 0.05); blue indicates positive correlations and red indicates negative. C, carbon (%); N, nitrogen (%); P, phosphorus (%); C:N, the ratio of carbon to nitrogen; N:P, the ratio of nitrogen to phosphorus; C:P, the ratio of carbon to phosphorus.

The plant N and P concentrations and C:N:P stoichiometry were significantly correlated with the soil C, N, and P concentrations and C:N:P stoichiometry (Fig. 3, *P* < 0.05). The correlation analysis showed that litter N and P were significantly positively correlated with soil C, soil N, soil P, and the soil C:P and soil N:P ratios. Root N was significantly positively correlated with soil C, soil N, and the soil C:P and soil N:P ratios. The N:P ratio of the soil and roots showed a significant positive correlation with the C:P ratio of the soil and roots and a significant negative correlation with the C:N ratio of the soil and roots. The N:P ratio of the leaves and roots were significantly positively correlated with the soil N:P ratio.

## Discussion

### Effects of desertification on plant C, N, and P concentrations and C:N:P stoichiometry

Grassland desertification has a significant influence on plant C, N, and P concentrations and C:N:P stoichiometry. Our results indicated that the leaf N concentration was higher in the VSD stage (mobile sand) than in the other desertification stages. These results were consistent with the variation in leaf N along the sandy grassland restoration process^35^. *Agriophyllum squarrosum* (annual forb) was the dominant plant in the VSD stage (mobile sand). Pioneer plant species (*A*. *squarrosum*) maintained higher plant N concentrations when growing in mobile sand with poor nutrients^35^. Variations in C, N and P stoichiometric relations were observed among the different plant tissues^22,36^. Leaves tended to have greater N and P concentrations and thus lower C:N and C:P ratios than litter and roots across all desertification stages. This revealed that more nutrients are redistributed to the aboveground plant tissue parts to support shoot regrowth. The variation in plant C:N:P stoichiometry resulted from the changes in different plant tissue C, N, and P concentrations. Furthermore, the C:N:P stoichiometry of different plant tissues is linked to the chemical composition of the different plant tissues.

We found that the plants in the PD, LD and VSD stage showed higher leaf N:P levels than those in the MD and SD stages. The plant communities in the PD and LD stages are rich with leguminous families and other N_2_-fixing plants. When the plant community changes due to desertification into an *A*. *squarrosum*-dominated community, this changes the physical properties and available nutrients in the soil, consequently inhibiting plant nutrient absorption and utilization. In addition, the leaf C:P ratio ranged from 205 to 236 during the grassland desertification process. Our findings were lower than the presumed leaf C:P threshold (250:1) required for the efficient growth of P-rich herbivores feeding on comparably C-rich plants^37^. The leaf C:N ratio (15.1–24.8) was in agreement with the leaf C:N ratio (17.9) across the three grasslands of China^18^. The leaf C (41.3%), N (2.24%), leaf C:P (222) and N:P (12) ratios in our study were lower than those of several species on the Loess Plateau (43.8%, 2.41%, 312, 15.4, respectively), and leaf C:P (232) and N:P ratios (12.7) found for global flora^37^, while leaf P (0.188%) was higher than that of Loess Plateau (0.16%)^38^. This suggests that the lower leaf C:P and N:P ratios compared with the Loess Plateau are likely caused by low leaf C and N, and high leaf P. Plant P is generally related to soil P, and the high leaf P may be due to the high soil P content^17^.

### Effects of desertification on soil C, N, and P concentrations and C:N:P stoichiometry

It has been demonstrated that grassland desertification changes soil nutrients^39–41^ and leads to alterations in soil nutrient stoichiometric relations^23,40^. Along the grassland desertification gradient in our study area, there were significant variations in soil nutrients (soil C, N, and P) and C:N:P stoichiometry (soil N:P and C:P ratios), implying an influence of grassland desertification on soil nutrients and stoichiometry. These successional changes in the C, N, and P concentrations and C:N:P stoichiometry of the soil support the hypothesis that soil nutrient cycles and thus plant growth are influenced by grassland desertification. The decreases in soil C, N and P concentrations following grassland desertification were similar to those found by Zuo *et al*.^3^ in Horqin Sandy Land. The decreasing soil N and C from the PD stage to the VSD stage suggests that grassland desertification leads to a loss of soil organic C and N. The loss of soil organic C and N demonstrated substantial environmental degradation over the process of grassland desertification. The decrease in vegetation coverage and productivity would inevitably cause the loss of soil organic C and N with increasing desertification. Grassland desertification induces the release of greenhouse gases thereby loss of C and N from the soil into the atmosphere^11^.

Understanding changes in soil C:N:P stoichiometry following increasing desertification is important for estimating the content of soil nutrients and sustainable development in desert grassland ecosystems. The soil N:P and C:P ratios decreased significantly over the process of grassland desertification. The soil C:P (3.6) and N:P (0.3) ratios in the desert grassland were lower than the average ratios for China and worldwide^23,42^. The soil C, N and P concentrations only account for 5.7%, 6.3% and 48% of those found by Tian *et al*.^23^ in China’s soils (2.46%, 0.19% and 0.08%, respectively). The soil N:P ratio and N concentration in this study were lower than those estimated at the global^42^ and regional scale^23^, which suggests N limitation occurs in desert grasslands. The values we found in this study were similar to the soil C:N ratios (11.1) that were found by Yang *et al*.^43^ in topsoil of China’s grasslands but lower than those estimated by Cleveland and Liptzin^42^ at the global scale and Tian *et al*.^23^ in China. The soil C:N ratio was relatively consistent across the different desertification stages. Despite the diversity of soil properties, structural complexity and spatial heterogeneity, the soil C:N ratio is relatively consistent across various terrestrial ecosystem types at the global scale^42^. The constrained soil C:N ratio is consistent with the stoichiometric principles that soil organic matter formation requires N and other nutrients in a relatively fixed ratio with C and highlights that soil C and N are tightly coupled in natural ecosystems^44^.

### The plant-soil relationships of C, N, and P concentrations and C:N:P stoichiometry

In natural ecosystems, nutrient elements are cycled between the soil and plants^22^. The strong correlations among C, N and P in the soil or in plants have been demonstrated by many studies^24,45,46^, while few studies have focused on how C, N, and P concentrations and C:N:P stoichiometry in soil are related to C, N, and P concentrations and C:N:P stoichiometry in plants^47,48^. There was a significant positive correlation between the soil N:P and leaf N:P ratios in desert grasslands, which was consistent with the relationship between soil N:P and leaf N:P ratios in a subtropical region^49^. The relationships between plant and soil stoichiometry are most likely driven by two mechanisms. On the one hand, plant nutrients are limited by soil nutrient availability^19,50^. On the other hand, these relationships were confirmed to be affected by nutrient re-translocation between soil and plants. Fife *et al*.^51^ found a similar re-translocation pattern for leaf N and P among different plant species. Soil nutrient stoichiometry is tightly linked with plant nutrient stoichiometry in the semiarid grassland ecosystem. The N:P ratio of the plant tissue (leaves and roots) and soil were positively correlated with the C:P ratio of the plant tissue (leaves and roots) and soil and negatively correlated with the C:N ratio of the plant tissue (leaves and roots) and soil, which is consistent with the results from a previous study by Bell *et al*.^52^ in a semiarid grassland ecosystem. Soil and leaf C:N ratios were positively correlated with the soil and leaf C:P ratios^52^. Our results suggest that nutrient concentrations and stoichiometry in soil and plants are tightly linked in desert grasslands. The decline in plant nutrients is also associated with soil nutrient loss from the grassland ecosystem over the process of desertification.

## Materials and Methods

### Study site

The study site was located in Yanchi County, Ningxia, China (37°04′-38°10′N and 106°30′-107°41′E, elevation approximately 1450 m), which is located at the southwestern margin of the Mu Us Desert. This region has a temperate and semiarid climate. The annual precipitation is 280 mm, with approximately 70% occurring during the June to September period. The annual potential pan evaporation is approximately 2710 mm, which is equivalent to more than nine times the annual precipitation. The annual temperature (MAT) is 8.1 °C, with monthly temperatures ranging from −8.7 °C to 22.4 °C. The annual wind speed is 2.8 m·s^−1^, and the prevailing winds are mainly northwest in April and May. Wind erosion often occurs from April to mid-June before the rainy season starts^53^. At the study site, the main soil types are arenosols of quartisamment, which is barren with a loose structure and vulnerable to wind erosion^34,54^. The vegetation is dominated by *Agriophyllum squarrosum*, *Salsola collina*, *Corispermum hyssopifolium*, *Artemisia scoparia*, *Pennisetum centrasiaticum*, *Aneurolepidium dasystachys*, *Cleistogenes gracilis*, and *Lespedeza potaninii*.

### Desertification degree

There are many criteria for assessing the degree of grassland desertification^55,56^. In terms of the types and degrees of grassland desertification offered by Li *et al*.^56^ and our investigation, five types of grassland in different desertification stages can be identified: (i) potential, (ii) light, (iii) moderate, (iv) severe and (v) very severe desertification. Overgrazing is one of the primary causes of grassland degradation with different desertification degrees. A space-for-time approach was used in selecting experiment sites, and the five desertification stages were represented by the existing grassland stands. The potential desertification stage (PD, regarded as control) is non-degraded grassland, with a vegetation cover of more than 70%. The light desertification stage (LD) is characterized by fixed sand. Mobile sand composes from 1 to 2% of the total grassland area, and vegetation cover covers from 50 to 70%. The moderate desertification stage (MD) is characterized by semi-fixed sand. Mobile sand occupies approximately 5–20% of the grassland area, and vegetation cover composes from 30 to 50%. The severe desertification stage (SD) is characterized by semi-shifting sand dunes. Mobile sand accounts for 20–50% of the grassland area, and vegetation cover is between 10 and 30%. The very severe desertification stage (VSD) is characterized by shifting sand dunes, mobile sand occupies more than 50% of the total area, and vegetation cover is reduced to less than 10%. The original condition of these desertified sites was entirely grassland with similar topography and soil type. Therefore, the sites representing the five desertification stages only represented different desertification degrees and were otherwise comparable to one another.

### Vegetation and soil sampling

Fifteen sites (50 m × 50 m) with similar topography representing the five different desertification stages were established in August 2013. Ten randomly placed quadrats (1 × 1 m^2^) were established for vegetation sampling at each site. In each quadrat, vegetation was harvested according to species at ground level, and residual standing litter was hand sorted and added to the raked litter. The harvested plants from each quadrat were separated into stems and leaves and then oven dried at 70 °C for 48 h to a constant weight. Within each quadrat, litter (vegetation produced in previous years) was first removed from the quadrats by hand raking and retaining. Fine, fragmented and partially decomposed litter (humus) lying on the soil surface was not included since it was mixed with mineral soil and could not be separated. Within each quadrat, roots were collected at 0–40 cm in three soil cores (diameter 9 cm). In the laboratory, the soil was washed away from the roots, and then the roots were oven dried at 70 °C for 48 h.

In every quadrat, three soil samples were collected from 0–20 cm depths by taking soil cores, which were then mixed into one compound sample. Each soil sample was sieved through a 2 mm mesh. The leaf, litter, root and soil samples from two quadrats were combined, which created five replicate samples for each plot. The leaf, litter, root and soil samples were ground to homogeneity with a ball mill for C, N and P measurements.

### Sample analysis

The concentrations of C, N, and P (percentage dry mass, %) were determined for all plant tissue samples. Analysis of C and N concentrations was performed on an elemental analyser (multi N/C 3100 TOC, Germany). The tissue P concentration was determined by the molybdenum blue colorimetric method with a UV/visible spectrophotometer (UV-2450/2550, Japan). Soil P determination followed the same basic methods as plant tissue analyses. The soil organic C concentration was analysed by the Walkley-Black modified acid-dichromate FeSO_4_ titration method^57^, and the soil N concentration was determined using the Kjeldahl acid-digestion method. Soil and plant C, N, and P concentrations were expressed on a dry weight basis. C:N:P stoichiometry of the plant tissue and soil was calculated on a mass basis.

### Statistical analysis

One-way analysis of variance (ANOVA) was used to determine the differences in nutrients and stoichiometry of the plants and soil among the different desertification stages. Homogeneity of variance and least significant difference (LSD) tests were conducted following the ANOVA to determine the significance of the differences among treatments at *P* < 0.05. Analysis of variance (ANOVA) was performed using SPSS software (SPSS Inc., USA). Pearson correlations were calculated to determine how the nutrients and stoichiometry of the plants and soil components were correlated during grassland desertification; this correlation analysis was conducted with the “cor6plot” package in R version 3.2.4 (R Core Team 2016).

## Data Availability

The dataset generated during the current study is available from the corresponding author on reasonable request.
